# Some Common Causes of Pain in the Foot.—I

**Published:** 1910-09-17

**Authors:** 


### ORTHOPÆDICS.

### SOME COMMON CAUSES OF PAIN IN THE FOOT.—I.

The deformities which the orthopædist is called upon to treat are not usually looked upon as being painful lesions. In fact, most text-books and the majority of teachers entirely ignore the fact that it is sometimes as important to treat the pain of a simple deformity as it is to correct the deformity itself. The reason for this general silence upon the subject of pain is probably that the majority of cases which come into hospital or reach the specialist are advanced cases. In such the pain, whatever there is of it, is entirely overshadowed by the deformity. Objective symptoms are treated and the subjective ones disregarded, except when the patient insists upon being relieved of what may be a constant inconvenience to him. In early cases, however, subjective sensations are by no means uncommon, and pain, in some instances sharp, worrying, and excruciating may make the patient's life a misery. As the deformity advances the ligaments and tissues get stretched, relax, and accustomed to the change of relations with nerves or vessels, and in consequence the pain becomes less and less aggravating. But it is highly desirable to control the lesions before that stage is reached. The practitioner who attempts to treat orthopædic cases must ever bear in mind that the best results are obtained by early interference and early attention. Such interference need not necessarily be operative, but it should be thorough and systematic.

With this preliminary introduction we may proceed directly to deal with the more common types of pain that the practitioner has to deal with in cases of tarsal and metatarsal lesions. By far the most common variety is the pain resulting from flat-foot.

### Pain in Flat-foot.

This is especially well marked in the early stages, and, indeed, it may be the only sign to lead the examining practitioner to suspect a flattening of the tarsal arch. The pain is of varying intensity and character. Probably the most common form is a dull ache which is felt chiefly in the calf muscles when the patient is standing, but which may be complained of even when the limb is in a resting condition. Equally common is radiating pain in the foot itself, more commonly felt on the dorsal aspect. Both types may be very severe, and, in young subjects, are apt to be classed as rheumatic or "growing pains." In all cases where pains in the foot or leg are complained of a careful examination of the arches should be made, and a ferric chloride print of the soles of the feet should be obtained at the first examination and compared with one taken later on. By that means very slight flattening is easily to be detected, and can be summarily treated. In rare cases the pain of flat-foot is felt in the ankle joint, and may be so severe as to cause a suspicion of tubercular disease. The diagnosis, however, is readily made with care, since the pain in such instances is only complained of when the patient has been standing or walking about, is never experienced when the patient is resting, and is easily subdued by appropriate treatment.

Still a further variety is knee pain, which usually, rightly enough, attracts attention to the hip-joint, but rarely leads to a close examination of the foot. Pain above the knee is very rarely experienced as a result in uncomplicated flat-foot cases, and, when present, is usually due to scoliosis or sacro-iliac changes, with which we will deal later. The pain in a typical flat-foot case is more or less constant, and diffused over a wide area. It is only rarely that one comes across a case in which it is strictly localised to the medio-tarsal joints or to the dorsum. In a few cases there is associated tenderness to pressure over the painful areas, especially along the course of the internal plantar nerve.

Treatment.

The only radical treatment of the pain in cases of flat-foot is to treat the initial lesion. Salicylates, aspirin and the various anodynes may produce temporary comfort, but as the pain depends on anatomical defect, it can only be properly relieved by treating the lesion which has produced that defect. Massage may relieve the pain considerably in some cases: in others it produces little effect: in any case it must be properly carried out by an experienced masseur, for mere unskilful rubbing of the foot or leg will not do. Exercises as usually prescribed in cases of flat-foot must be strictly avoided. In ordinary cases of advanced flat-foot they do no good, but at least do not make matters worse, but in early cases they are mischievous. It is inconceivable that a few minutes' daily abnormal and forced movements will be able to produce what the constant and continual ordinary action of the muscles, as in walking and standing, cannot achieve. Nevertheless, exercises are still prescribed by many practitioners, and are regularly given in the text-books. The proper treatment of flat-foot calls for a more detailed description than can be here given. With regard to the pain that accompanies the condition, it need merely be mentioned that this will in most cases readily yield to such treatment as is required in ordinary cases. A properly moulded inset must be worn, and the practitioner must bear in mind that he will achieve the best results and win the confidence of his patients by making these insets himself. For that purpose a plaster cast of the patient's foot is required, from which the cork, leather, or celluloid inset may be moulded. Such insets are easily worn in ordinary boots or shoes, and when properly prepared will rid the patient of all his pain. In many cases the results are striking and the patients proportionately grateful. To be effective, however, the practitioner must mould the inset himself, or at least see that it is properly moulded and closely superintend its construction. Nothing is more absurd than to let the instrument-maker select a ready-made inset from his pile and fit it to a patient, or to let the latter buy the first "brace" he sees in a shop-window and apply it himself.

There is a variety of foot pain, associated with a marked tendency towards flattening of the arch, met with in old people. This is most commonly a form of tabetic pain, though it can scarcely be designated as "lightning pain." It is felt over the dorsum of the foot, chiefly on the inner side, and along the outside of the leg, over the peronei; more rarely it is complained of behind the ankle. This variety of pain is persistent, and may even be associated with a Charcot's joint at the ankle or knee, obscuring the diagnosis of this always painless lesion. In such cases it must be noted that the pain is muscular, not felt over the swollen and flail-like joint, and analogous to pains in other parts which the patient may experience at the same time. In all cases of pain associated with flattening of the arch of the foot occurring in old subjects, the ankle and knee reflexes should be tested and the pupil reaction carefully investigated, as tabes should always be suspected. The treatment is usually very unsatisfactory; only morphia in moderately large doses appears to control the pain, though doses of ammonium chloride are worth a trial. As the disease progresses the pains lessen, and ultimately vanish altogether. Wearing a pad or inset does not appear to have any effect in minimising the pains of a tabetic subject with flat foot.

The foot pain complained of by pregnant women is usually due to a flattening of the arch of the foot, and is readily relieved by appropriate insets.

(*To be continued.*)

